# Influential Factors on Collective Anxiety of Online Topic-Based Communities

**DOI:** 10.3389/fpsyg.2021.740065

**Published:** 2021-10-05

**Authors:** Yi Yang, Na Ta, Kaiyu Li, Fang Jiao, Baijing Hu, Zhanghao Li

**Affiliations:** ^1^School of Chinese Culture and Communication, Beijing International Studies University, Beijing, China; ^2^School of Journalism and Communication, Renmin University of China, Beijing, China; ^3^Department of Computer Science and Technology, Tsinghua University, Beijing, China; ^4^School of Journalism and Communication, The Chinese University of Hong Kong, Hong Kong, SAR China; ^5^Computational Communication Research Center, Beijing Normal University, Zhuhai, China; ^6^School of Journalism and Communication, Beijing Normal University, Beijing, China

**Keywords:** social media, affective polarization, collective anxiety, topic-based community, Weibo, neural network model

## Abstract

**Background:** Under the uncertainty led by the decentralized information on social media, people seek homogeneity in either opinions or affection to establish group identity to better understand the information. This also means they are easily polarized, not only ideologically but also in their actions. Affective polarization is the emotional tendency for people to show animosity toward opposing partisans while seeking homogeneity from fellow partisans. Much research into online affective polarization has focused on quantifying anxiety at an individual level while neglecting that on a collective basis. Therefore, this paper examined the polarization of collective anxiety in topic-based communities on Weibo.

**Methods:** We aim to interpret correlations between collective anxiety online and topic characteristics, user competence, as well as the proportion of influencers of Weibo topic-based communities. Our neural networks model and statistical analysis were based on 200 communities with 403,380 personal accounts and 1,012,830 messages.

**Results:** Collective anxiety levels are correlated to (1) the extent to which a topic captures public interest, (2) how community members articulate topics on social network platforms, and (3) the ratio of influencers in the community. Specifically, people’s conflicting perceptions and articulations of topics might increase collective anxiety, while the extent to which a topic is of the public interest and the number of influencers engaged in a topic account for any decline in its ranking. Furthermore, familiarity with a topic does not help predict collective anxiety levels. There are no significant links between community size or interactivity dynamics and the level of collective anxiety in the topic-based community. Our computational model has 85.00% precision and 87.00% recall.

**Conclusion:** This study found the collective anxiety augment due to topic proximities to public interest and members’ lack of declarative knowledge on topics, while to decline with an increasing portion of online influencers. These findings indicate that collective anxiety is induced due to a lack of credibility. Also, the amount of conflicting information shared by different people places them in a state of flux. Therefore, a community with more influencers may be more likely to experience anxiety polarization, bringing forth the issue of layered information and inequality.

## Introduction

The exact impact of social media on mass polarization, especially affective polarization, is still up for debate. Polarization refers to the process in which individuals seek homogeneity at information, affective, and identity levels to strengthen their original tendencies. Extensive scholarly literature has considered polarization among deliberative groups. Some scholars showed how partisan moral convictions can increase an individual’s willingness to block friends who support out-party members on social media (Garrett and Bankert, 2020). Others, by contrast, examined how this kind of polarization can be relieved by a framework of national identity, which reduces the distrust between different parties (Levendusky, 2018). A significant facet of the issue is affective polarization, which is also used as a mechanism of discord. Some studies have looked at the relationship between deliberation and affective polarization. For example, there was a reciprocal relationship between discussion with a higher proportion of like-minded partners and affective polarization (Hutchens et al., 2019), whereas people’s exposure to uncivil online discussion did not affect attitude polarization (Hwang et al., 2014).

The emergence of high-choice media is reckoned as a realistic cross-country background for political deliberations, either online or offline (Iyengar et al., 2012; Lelkes and Westwood, 2017). Therefore, most existing studies have focused on deliberation and affective polarization. Some scholars have examined how people’s selective exposure correlates with their emotions. For example, the audience’ exposure to pro-party television sources might strengthen their anger and fear of out-party members, negatively impacting deliberative democracy (Lu and Lee, 2019). Similarly, negative political advertisement exposure, combined with more diversified media sources about presidential candidates, drove people toward liking their preferred candidate (Lau et al., 2017). Polarization was first understood as the “un-averaging” of opinions or judgments of individuals in a group, especially after group discussions (Moscovici and Zavalloni, 1969; Myers and Lamm, 1975). Since the 1970s, studies into politics have begun to focus on the relationship between partisanship, psychological involvement, and the formation of polarization (Pierce, 1970). During the past decade, the relationship between polarization and collective and affective practice has aroused scholars’ interest (Garrett et al., 2014; Iyengar et al., 2019), which is the focus of our study. Although many scholars have shown a particular interest in the effect of the media environment on polarization, controversy remains over the extent to which media can realize its polarization potential. This controversy has been intensified with the rise of social media, when seeking homogeneity also functions as a significant social network principle. Several researchers demonstrated that the consumption of social media was crucial in shaping political mood by creating misleading perceptions (Shahini-Hoxhaj, 2018). Conversely, others believed that it is a person’s prior opinion, rather than their information–choice behavior, that motivates polarization. Therefore, the polarizing effects of digital media tended to be more conspicuous when the immediate political context involved a highly salient divisive issue (Leeper, 2014; Lee, 2016).

All these findings and arguments share common implications. First, most scholars use case studies to empirically prove or disprove the effect of polarization rather than showing the concrete procedure of people’s tendency to shift through specific models. Second, there is a broad consensus that, except for opinions and information-seeking behaviors, polarization is also closely related to mass public emotional states. Although some scholars touch on positive or negative sentiments, they ignore discrete emotions such as enthusiasm, fear, anger, and anxiety. Each emotion is actually unique and mediates a different deliberation process (Nabi, 2010; Lu and Lee, 2019). For instance, some scholars reported that enthusiasm about out-group candidates might have a unique ability to disrupt affective polarization (McLaughlin et al., 2020). The origins of these discrete emotions, as well as their correlation with the mediating roles of social media and users’ personal influence, are less clear, however. Moreover, to the best of our knowledge, much of the literature regards polarization as a static state or the result of deliberation. Few scholars have explicitly illustrated how polarization is formed when emotion plays an “iterated game” (Sunstein, 2002) with the intervention of social media.

To address these gaps, this paper proposes a machine-learning model that computes the alterations to collective anxiety, a discrete emotion at the community level. Explication of anxiety occurs *via* homophily on social network platforms. Our research concerns how topic-based communities affect collective anxiety on Weibo and then create affective polarization. This project aims to comprehend correlations between Weibo collective anxiety and core determinants of topic-based communities, specifically topic aspects, users’ competence levels, and proportion of influencers.

### Topic-Based Community and Its Underlying Affective Polarization

A topic-based community revolves around subjects of common interest, delineated by hashtags. Two core features govern the topic-based community. First, it is composed of a fleeting presence of the public (Blackman, 2012). When the issue is trending or interesting, people will emerge. However, upon solving the conundrum or losing interest in it, the community effectively disintegrates. A topic-based community is, hence, devoid of established social norms and group identities; in this type of milieu, members are easily manipulated or taken advantage of. Second, a topic-based community features high content visibility, unless under special privacy settings. Irrespective of whether people follow each other or not, all topic-based community users can easily see the posts, reposts, and comments or others. As people naturally gravitate toward other people with similar emotional proclivities (González-Ibáñez and Shah, 2010), they can find like-minded collaborators more easily as a result of the high content visibility that exists within topic-based communities (Bessi et al., 2016; Wojcieszak et al., 2020). Consequently, group think, resulting from emotional contagion, is highly probable.

These two features harken Le Bon’s tenet of the crowd; writers posit that social-network sites like Weibo are the quintessential fertile breeding grounds for establishing Le Bon’s crowd proposition, the concept of which has been found related to collective emotions in both offline and online spaces. For example, Hopkins et al. (2016) investigated the formulation of positive emotions in crowds during the Hindu pilgrimage festival in north India. Stage (2013) also found Le Bon’s concept can help understand affective relationships on social media from the perspective of crowd psychology. An entity formulated by similar thoughts, rather than physical gatherings, the crowd, as per Le Bon, is a process of deindividuation that is impelled by emotional suggestion and affective contagion (Bon, 2002; Vilanova et al., 2017). During this process, bellwethers can grab control over the crowd by the long-distance application of symbols and media that makes crowd members adhere to contextual protocol, as opposed to fixed social norms or identity guidelines (Bon, 2002).

A plethora of scholarship has confirmed the insights of Le Bon’s crowd theory for social media research. Especially for online circumstances, Le Bon’s crowd theorem can emphasize how emerging online technologies change the dynamics of publics and collectives (Olofsson, 2010; Stage, 2013). The writers agree, in principle, with their assertion. From both the temporality of the members and content visibility, a topic-based community constitutes an online version of Le Bon’s crowd. Concerning the former, a topic-based community is not fixed, and is, instead, a fluid entity. This is also the fundamental community genre of social networking sites (Myers et al., 2014). With respect to the latter, the content visibility and traceability of the topic-based community proffers a solid pathway to actuate Le Bon’s emotional contagion. The authors argue that affective polarization is a potential concern of Le Bon’s crowd paradigm. More specifically, Le Bon describes the link between crowd actions and the overarching, yet transient collective emotion. Whereas crowd members are especially prone to blindly follow others due to emotion, these behaviors then reinforce the collective emotion (Bon, 2002). Therefore, the authors postulate that Le Bon’s concept of the crowd enhanced our comprehension of the topic-based community as a fleeting online entity. Furthermore, his depiction of the attributes of collective emotion also prognosticates online affective polarization of this contemporary Internet era. Henceforth, the core question of this study is pertinent to the structure of the online topic-based community itself and its correlation with the collection emotions, particularly with respect to collective anxiety polarization. Prior to asserting any additional research hypotheses, it is mandatory to explicitly define the term, collective emotions and explicate the reason for exploration of their polarization on social-network sites like Weibo.

The authors defined collective emotion as an affective trend formulated by the individual emotions of community members *via* social contagion. We particularly emphasized three aspects of collective emotion. First and intuitively, collective emotion is an emotional trend shared by large numbers of individuals (Bar-Tal et al., 2007). Therefore, it will be more accurate to aggregate collective emotions based on the data of large-scale community members. Second, a collective emotion is motivated by shared experiences among community members, rather than a sum of individual reactions to his or her private life (Schweitzer and Garcia, 2010). That means emotional contagion and iteration in social networks play a pivotal role in its formation. Third, the proliferation of social media has added nuance to collective emotion, as it creates a porous boundary between public and private spaces (Dahlgren, 2005), causing one-to-many mass communications and one-to-one interpersonal communications to mix together. Hence, the flow between and convergence of individual emotions and collective emotions have become easier and more frequent. As such, to those members of subgroups, social contagion is more likely to occur, and collective emotion is more likely to emerge. To encapsulate this trend, we aggregated individual emotional levels and created an interactive spiraling protocol; we also showcased anxiety as a collective emotion. Juxtaposed with other emotions, anxiety is a profound, comprehensive phenomenon. Anxiety is germane to the unease materializing from a challenge to one’s values and actions as well as to more explicitly strenuous circumstances (May, 2015), like food safety crises (Hadley and Patil, 2008), or nuclear power plant accidents (Takebayashi et al., 2017). When the safety index of societal members falls, anxiety results. For our study, each member interacts with online neighbors to gradually shape his specific anxiety level, one degree at a time, until the kth degree neighbor. Collective anxiety of a topic-based community is an amalgamation of all members’ anxiety levels.

Social networking site dominance has new pertinence to the polarization of collective emotion. Polarization is usually realized through a psychological mechanism called homophily. When countenanced with positive data, people typically reveal positive feelings. They either discover people with similar thoughts (Festinger, 1954) or search for information about a valued topic-the information-seeking bias (Meppelink et al., 2019). Thus, this tenet explains homophily, the key pathway enabling the polarization of public discourse culture (Colleoni et al., 2014). Homophily asserts that the interaction of beliefs, attitudes, and actions of people with other individuals creates a diffusion-related function (Sunstein, 2009; Borgatti et al., 2013) and the formation of subgroups. Subgroup members connect with each other more, so they are more likely to become infected. What social contagion entails is that emotions, thoughts, or behaviors transition from the initiator to the recipient, often unbeknownst to the latter (Levy and Nail, 1993). Consequently, the opinions and emotional tendencies of subgroup members tend to be stronger, compared to the time of subgroup formation, in turn, forming the thoughts and affective polarization (Mäs et al., 2013). For the social networking site, the previously referenced homophily is promoted by the high content visibility. Pursuant to our research, the interactive nature of the topic-based community empowers the user to follow particular accounts irrespective of owner approval (Colleoni et al., 2014). This aspect expedites communication with key opinion leaders (KOLs), whose primary method of influence is due to the volume of non-reciprocal follows (Veirman et al., 2017). This also enables more traceability of information flow so that users can better grasp the characteristics of certain events. Thus, users can more easily discover cohorts who share similar emotional patterns, affective status components, or impacts by KOLs, either actively or passively. Within a topic-based community, it is more probable that affective polarization will transpire.

The literature on technological and affective affordances has offered another insightful lens to explain topic-based community members’ tendency to be affectively polarized. Affordance relates to a user’s cognitive heuristics on the use of technological objects. Accordingly, affective affordance refers to the mechanisms of an object or an environmental element that can transmit and collect people’s contextual affective meanings (Zhao et al., 2013). Scholars propose a cogent sketch of the recursive dynamics between people’s intent to use technology and their affective and emotional perception of its affordances. Specifically, technologies may help transmit or collect users’ affective meanings in a specific context, and further drive their emotional perception of their uses. These emotional experiences will, in turn, exert strong influences on their intentions to use (Shin, 2016). For instance, Shin and Hwang (2020) suggest that blockchain mechanisms make it possible for multiple users to interact, transparentizing the organization process and its relevant data. Such affordances will decode users’ experience of transparency and trust. On the other hand, Shin and Kim (2015) suggest that the more users find it pleasant to communicate with others using supporting technologies, the more intention they will have to use these technologies. In other words, they have successfully proven that users’ perceived sociability is positively associated with their intended technology use.

Unlike the psychological homophily mechanism approach, these studies highlight users’ affective experiences and perceptions in the space created by technologies. This affordance approach provides insights into our research.

First, the above-mentioned non-reciprocal following mechanism and the hyper-connected, traceable Weibo content may strongly influence topic-based community members’ perceptions of collaboration and trust. This kind of user experience and perception could motivate networked emotion and what we called social contagion, which paves the way for affective polarization.

Second, the essence of the porosity of the Weibo platform is what Shin and Kim (2015) call “socio-usability.” Weibo is not only viewed as a technology environment for posting and commenting, but it is also perceived as an online space for interactions. This socio-usability accounts for Weibo’s potential of driving shared affections and emotions, which might lead to affective polarization in extreme cases.

To sum it all up, we proposed topic-based communities and their underlying social polarization as a comprehensive theoretical framework. Our research questions are as follows:

RQ1: What are the elements of the topic-based community? How can we effectively comprehend the specific determinants such as the specific features of the topics, community size, dynamics of interactivity, popularity, and the competency levels of the members?

RQ2: What is the correlation between the aforementioned characteristics of Weibo topic-based communities and affective polarization, specifically, anxiety polarization?

To answer these research questions, we further propose following hypotheses.

## Materials and Methods

### Hypotheses

Public interest plays a pivotal role in the formation of topic-based communities. The closer a topic is to the pulse of public interest, the more likely it is to wield a plethora of emotional impacts. In such an era with high complexity and mobility, what is deemed public interest has no overarching definition, but it is frequently determined contextually (Johnston, 2017). Following our discussion on socio-usability in the literature review, we can assert that the affordance of Weibo is to make such a contextual public interest highly debatable. Therefore, we use user preferences to operationalize the publicness of a topic. Topic-based communities are rapidly constructed entities for social networking sites based on user preferences (Kardara et al., 2012). In our study, the preferences are measured by the topic-based community’s size. Numerous studies indicate that collective anxiety will change with the issue’s relevance to public interests. For instance, researchers agglomerated data concerning collective anxiety levels on 51 topics related to the 2011 Tohoku earthquake and the Fukushima nuclear power plant leak. Only for subjects involving the two catastrophes did the anxiety level of display a marked level of change. The reason was that the public was familiar with these two issues that are directly correlated to the public interest (Nakayachi et al., 2015). Those topics most germane to public interest typically compel collective anxiety. Researchers have proven that, during the COVID-19 epidemic period, compared with other topics such as employment conditions, government initiatives, and traveling, subjects like self-protection and physical symptoms align better with public anxiety (Jo et al., 2020). Prior research impels us to be cognizant of the link between topic characteristics and collective anxiety of topic-based communities. Hence, we propose our first hypothesis:

H1: The topic’s proximity to prevailing social issues heightens the anxiety levels of the topic-based community.

H1a: With increased familiarity with the topic, members tend to display higher levels of collective anxiety.

H1b: Higher levels of collective anxiety will result from topics aligned with public interest.

Since H1 details the connection between relevance of topics to social matters and topic-based community anxiety levels, further research must detail how members can lower their collective anxiety. Social media users are exposed to information with various types and diversified functions. Among them, knowledge is formulated when users integrate personal experience, values, background elements, and expert opinions for a functional framework to assess and integrate new information (Davenport, 2000). Since users within a topic-based community consistently fuse their own experiences into existing group knowledge parameters through community discussions, this constantly recalibrated know-how and community dialogue impacts anxiety (Santabárbara et al., 2021). With sufficient clarity of knowledge, the members can comprehend the events and also explicitly articulate their vantage points in community symposiums. This means they have declarative knowledge (Schraw, 2006). Following up on H1, we therefore propose our second hypothesis:

H2: Community members will display lower levels of anxiety as their declarative proficiency about particular topics increases.

A multitude of studies reflect that influencers affect polarization (Soares et al., 2018; Reinikainen et al., 2020; Sokolova and Kefi, 2020). Homophily drives this process, which includes attachments, values, and effective characteristics (Erickson, 1988). This clearly references, that in social settings, people with shared attributes will more likely forge connections (Lazarsfeld and Merton, 1978). Users and influencers also display this proclivity for homogeneity. When rationalizing persuasion, the former will fortify his original viewpoints. Thus, the latter is more persuasive in polarized groups (Berelson et al., 1954). Emotion is often the *de facto* strategy to impact the thoughts and actions of group members (Gross, 2008; Curiel, 2020). This overall phenomenon also occurs in social network platforms (Tyagi et al., 2020). However, users in a topic-based community, as compared with those in a classical digital community, are empowered with great interactive reactions, such as reposts and mentions of other users that contribute to the echo chamber (Pariser, 2011; Xu and Zhou, 2020). *Via* this process, opinions, relationships, and emotions are conveyed (Tan et al., 2011; Blevins et al., 2019). Consequently, the flexibility and dynamics of collection emotion are elevated due to the higher visibility and interactivity of topic-based communities. What then, is the correlation between influencers and the emotional polarization inherent in topic-based communities? Pursuant to our research, can these topic-based community influencers impact collective anxiety patterns? Our third hypothesis is as follows:

H3: With more influencers, a topic-based community will effectuate lower overall levels of anxiety fluctuation.

### Research Settings

The following study – combining self-reported anxiety scale results and big data analysis on Weibo – was conducted between March and May 2020 and was approved by the XXXX research ethics committee in XX, XXX on February 20, 2020.

Our research focused on topic-based communities on Weibo. Accommodating 78% of all netizens in China, Weibo allows its users to develop their networks, including both strong and weak ties, by following one another and posting, forwarding, and commenting on messages (Weibo Data Center, 2019). Users are labeled according to different levels of influence, such as real identity-verified accounts and experts/well-known bloggers; the latter being considered as opinion leaders in their own specialties. 99% of Weibo users are ordinary people, and 57.14% of them are daily active users (China Internet Network Information Center, 2017, 2019; Weibo Data Center, 2019). This means that most Weibo accounts belong to autonomous users who are independent from the elites of institutions, markets, and civil society. By sharing their attitudes and emotional states on Weibo messages discussing certain topics, users are considered to be members of the corresponding topic-based communities, which are identified by pairs of hashtags in messages, e.g., #GMO Food#. Therefore, a topic-based community refers to a hybrid of social and affiliation networks.

We focus on the collective-level affective polarization inferred from the affective status of individual users who make up a topic-based community. Organizational accounts are hereby excluded. The existing literature has shown that two linked users are likely to be homogeneous in opinions (Webster and Abramowitz, 2017) and sentiments (Tan et al., 2011). With the agency of these communities, information about a specific topic is spread widely and reinforces the community’s boundaries (Eliacik and Erdogan, 2018).

### Dataset

The data was taken from multiple topic-based communities on Sina Weibo and included individual users, their posts and reposts, and their comments concerning certain social topics as identified by enclosed hashtags. We randomly selected a 4-week observation window (September 1–28, 2018) and automatically retrieved openly accessible Weibo messages and the corresponding user profiles using a Python 3.6 program and the application program interface with Weibo’s authorization. Then, we constructed and selected the top 200 topic-based communities (referred to as WB200) in terms of the number of messages. The size of the 200 communities in the WB200 dataset varied from between 42 and 44,803 personal accounts, 37 and 39,852 messages and 0 to 68,748 comments. These communities covered the following topics: art, education, the entertainment industry, movies and TV series, sports, and public affairs. The basic characteristics of these communities are listed in Table 1. In coding the topic proximity and familiarity, we used Krippendorff’s alpha (Krippendorff, 2004) to check the reliability of two human coders. All units opened for analysis had alpha values above 0.90, implying a high degree of consensus between the two coders.

**Table 1:** Statistics for the selected 200 topic-based communities on Weibo (WB200).

	No. of communities		Avg/max/min no. of messages	Avg/max/min no. of users	Avg/max/min no. of comments
Topic fields	Art	20	1843/15727/37	1930/16120/57	926/8343/0
	Education	20	764/2736/44	839/3400/42	296/1624/0
	Entertain-ment	60	3388/39852/53	3855/44803/73	8612/68748/80
	Movies and TV	40	1155/6393/47	1251/6814/66	1243/9239/0
	Public Affairs	30	1488/8579/45	1725/9667/65	1599/9404/0
	Sports	30	399/1876/42	497/2356/49	524/5261/0
Topic charac-teristics	Familiar, proximate	11	1356/3345/96	3302/9216/206	3241/9404/0
	Familiar, distant	153	1963/39852/37	4392/89606/84	3870/68748/0
	Unfamiliar, proximate	18	1768/8579/45	4086/19334/130	1108/6969/0
	Unfamiliar, distant	18	726/2736/42	1704/6800/102	754/5621/0

### Computational Model for Collective Anxiety of Topic-Based Communities

**Figure 1:**
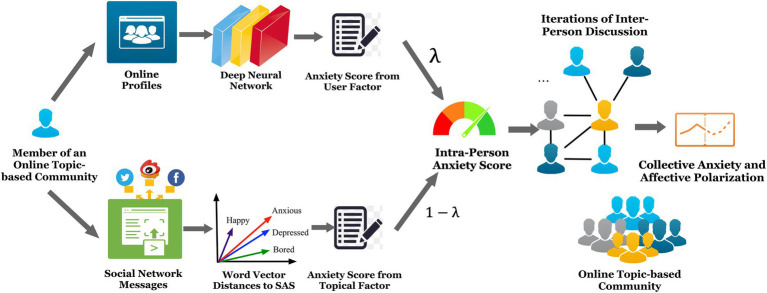
Framework of the computational model for collective anxiety evaluation.

Drawing on our previous study (Ta et al., 2020), we designed a cascading model to evaluate the shifts in the daily anxiety levels of the abovementioned 200 communities. For each community, we estimated the members’ personal anxiety levels by scoring their online profiles and posts. Then, we acquired their collective anxiety levels by iteratively simulating the process of online group communication. Figure 1 shows our computational model. A neural network was employed during this process, which helped us understand the connection between users’ online behaviors and expressions and their emotional status, given proper prior knowledge. Some pioneering research work used neural network models to explore similar research questions has enlightened our work. One typical example is Shin and Kim (2015), who prospectively used a neural network to predict users’ adoption behaviors. Their study breaks through the traditional linear regression method and offers us significant methodological guidance.

### Computing Individual Anxiety Scores

#### The User Factor

The characteristics of the Weibo users were partially embodied in their profiles (gender, education level, age, occupation, income, location, etc.). Therefore, we identified “profile” as the user factor in our model. We established a correlation between each member’s profile and their anxiety level to determine if the former could be used as a prediction for the latter. In practice, we first took the evaluated assessments of the Self-Rating Anxiety Scale (SAS)^1^ questionnaire of voluntary Weibo users and their profiles as the input. Second, we implemented a neural network model to gain insights into the correlations between the users’ profile features and their anxiety levels.

The training dataset (SAS360) for the neural network contained SAS results from users of Weibo who had volunteered to participate (*N*=360). The individual anxiety score ranges were divided into no anxiety (25–49), slight anxiety (50–59), medium anxiety (60–69), and extreme anxiety (70–100). For these users, the relationship between their anxiety scores and their Weibo profiles was established using their anonymized user IDs on Weibo.

After thorough training and tuning, the neural network used user profile data to assess each user’s anxiety score p, without asking each user to take the SAS test (see top left in Figure 1). In a threefold cross-validation of the SAS360 dataset, our prediction model had 85.00% precision and 87.00% recall.

#### The Topical Factor

In a topic-based community, members express their topic-specific thoughts and feelings *via* posts, reposts, and comments. We computed the topic-specific anxiety scores of each user and referred to these as the topical factor in our model as user messages flow through topic-induced connections within the group. In practice, the similarity between the keywords of the SAS questions and the Weibo messages was used as a major indicator. This was calculated using word vector and word embedding techniques.

Suppose two users posted two messages: w_1_=“I feel terrible, have too much work to do! #today#” and w_2_=“#today# It’s weekend! Life’s good.” The keywords extracted from the messages would be: “terrible” for w_1_ and “good” for w_2_. These words would then be converted to word vectors. Our Python program can then compute the cosine similarity between the word vectors of the keywords from the questions in the SAS (e.g., “nervous” and “afraid”) and the keywords in w_1_ and w_2_. As “terrible” is more similar to “nervous” and “afraid” than “good,” message w_1_ would indicate a higher anxiety level than w_2_. Hence, the user of w_1_ would have a higher topic-specific anxiety score than the user of w_2_.

For each community member, our model scored the similarity between each member’s messages and the SAS questions to a topical anxiety score m, with higher similarity indicating a higher level of topical anxiety (see bottom left in Figure 1).

#### Computing Individual Anxiety Scores

The initial individual anxiety scores of the community members u_i_ were acquired as follows:

$$a_{0}\left(u_{i}\right)\;=\;\lambda \;p\;+\;\left(1-\lambda \right)\;m$$ (1)

In this equation, p and m are normalized into the [0,1] range and λ is the parameter used to adjust the weights of the two factors (0≤λ≤1) so that 0≤a_0_(u_i_)≤1(Lee, 2016; Shahini-Hoxhaj, 2018; Lu and Lee, 2019). Furthermore, λ was platform-dependent and had to be carefully tuned according to the context.

### Computing Collective Anxiety Scores for Topic-Based Communities: A Cascading Method

In our proposed cascading model, each member’s anxiety level was impacted by neighbors at different proximities in the social network. The collective anxiety score was then acquired by gradually applying the inter-person contagion of anxiety *via* connections within the community (see right of Figure 1). In the k^th^ iteration of interaction, the impact from the k^th^-degree^2^ neighbors were transmitted to each user. Next, their anxiety levels were updated and re-evaluated, with the aggregation corresponding to the community’s collective anxiety. As the community’s deliberation persisted, we evaluated the collective anxiety level at different time points (e.g., on a daily basis) to determine if group polarization existed and how it developed.

Let Â_0_=[a_0_(u_1_), a_0_(u_2_), …, a_0_(u_n_)]^T^ (Bon, 2002; Eliacik and Erdogan, 2018) denote the initial vector of the community’s individual anxiety scores before diffusing anxiety between members u_1_, u_2_, …, u_n_. After applying the influence of the (k-1)^th^ degree connections to each member (k=1, 2, 3, …), the anxiety vector becomes.

$${Â}_{k}=\;{Â}_{k-1}\;+\;\;R\cdot \delta \left({Â}_{k-1}\right)$$ (2)

In other words, a community’s current anxiety level is based on the anxiety level formed in the previous round of diffusion, plus the interchange of anxiety between members in the current round of diffusion. In the formula, R is the relation matrix of users. If users u_i_ and u_j_ are connected *via* social media, items r_ij_ and r_ji_ in R are set to 1, otherwise, they are set to 0. Function δ(·) is the propagation power function that reflects the propagating influence of the anxiety level of u_i_ to its neighbors in each iteration. For an anxiety score x,

$$\delta \left(x\right)=\varepsilon e^{\left(x-\mathrm{UB}\right)/\mathrm{UB}}$$ (3)

In this equation, ε is a small positive real number and UB is the maximum individual anxiety score.

After k rounds, the collective anxiety score for community C becomes.

$$A\left(C\right)=norm\left(\Sigma_{i=1}{}^{n}\;{Â}_{k}\left[i\right]\right)$$ (4)

In this equation, Â_k_[i]=a_k_(u_i_) is the anxiety score for user u_i_ after the k^th^ iteration. The function norm(·) was used to activate A(C) into the [0,1] real number range. The closer score A(C) was to 1, the higher the level of anxiety in the community and vice versa. In this paper, we used the summation of each member’s anxiety score instead of the average. This was because the size of the community matters in terms of evaluating collective anxiety. Intuitively, a community of 1,000 members could have more influence on society than a community of 10 members given similar average individual anxiety scores.

### Time Series of Collective Anxiety and Evaluation of Affective Polarization

In our cascading model, we illustrate that the iterative process of emotional states is transmitted within a group, both as a cause and a result. To empirically determine whether a topic-based community’s emotional trend is polarized as online discussions continue, we observed the daily variance of a community’s collective anxiety level after its establishment. If the daily degree of collective anxiety showed a trend of incensement, collective anxiety was accumulated, hence the community’s demonstration of affective polarization towards a more anxious state.

### Detecting Declarative Knowledge in Weibo Messages

We first extracted the sources of information in each community (i.e., the personal accounts of all original posts in the reposting chains). We then labeled their credibility, with verified Weibo accounts endorsed by offline social institutions labeled as credible sources of information. Three trained coders manually coded the credibility of over 19,000 verified Weibo accounts in our dataset and had intercoder reliability of over 90.00%. Next, we extracted Weibo’s ideas and keywords using the automatic summarization algorithm, and then computed their cosine text similarity to the community’s topic.

Using the Chinese Readability Index Explorer (CRIE) tool (Sung et al., 2016), we then examined whether users were able to generate readable texts during online deliberations. Weibo messages were uploaded to CRIE, which returned the readability evaluation with the following formula: 4.53+0.01*[difficult words] − 0.86*[simple sentence ratio] − 1.45*[content word frequency in logarithmic]+0.02*[personal pronouns]. Combining all three criteria, we evaluated the declarative knowledge embedded in a Weibo message m:

$$\begin{array}{l} \mathrm{DK}\left(m\right)=a\cdot \mathrm{source credibility}+\;b\cdot \mathrm{text similarity}\\ +\;c\cdot \mathrm{text readability} \end{array}$$ (5)

In this equation, a, b and c are weight parameters. For community C, the declarative knowledge at a certain time point t was:

$$\mathrm{DK}\left(\mathrm{C,t}\right)=\Sigma m_{i}\mathrm{DK}\left(m_{i}\right),m_{i}\;\mathrm{was included in}\;C\;\mathrm{at}\;\mathrm{time}\;t$$ (6)

### Evaluating Community Members’ Media Influence

For each topic community, we picked out the yellow “V” personal accounts to get their ratio versus the total number of personal accounts. Then, we computed the daily variance of collective anxiety for each community within the observation time window. By doing so, we established the interrelationship between the proportion of influential members and the fluctuation of collective anxiety in a topic-based community.

## Results

### Validity of Our Computational Model

We first demonstrate that our computational model could effectively estimate the collective anxiety level (namely, extreme, high, medium, little, and no anxiety) of online topic-based communities. We trained five coders to rate the 200 communities in WB200 into five anxiety levels (no anxiety, little anxiety, medium anxiety, high anxiety, and extreme anxiety) and determined the ground truth using the majority principle. The collective anxiety scores of the 200 communities estimated by our model were also mapped into anxiety levels. We then compared the rating results of our model to the ground truth to test the validity of our model, i.e., how valid the model could compute that a community is at certain anxiety level. We used the widely used precision and recall metrics:

$$\mathrm{precision}=\mathrm{tp}/\left(\mathrm{tp}+\mathrm{fp}\right)$$ (7)

$$\mathrm{recall}=\mathrm{tp}/\left(\mathrm{tp}+\mathrm{fn}\right)$$ (8)

In these equations, tp (true positive), fp (false positive), fn (false negative) counted cases where the model’s rating was consistent with, more severe than, or less severe than the ground truth, respectively. Our model effectively rated the communities’ collective anxiety levels with more than 85.00% precision and more than 87.00% recall (Table 2).

**Table 2:** Empirical evaluation of the computational model’s effectiveness.

Topic fields	Art	Education	Entertainment	Movies and TV	Public affairs	Sports
Precision	0.82	0.91	0.81	0.81	0.88	0.85
Recall	0.92	0.99	0.93	0.89	0.97	0.94

### Evaluation Outcomes

*H1: Correlations between the topic of a community and the collective anxiety level.*

**Figure 2:**
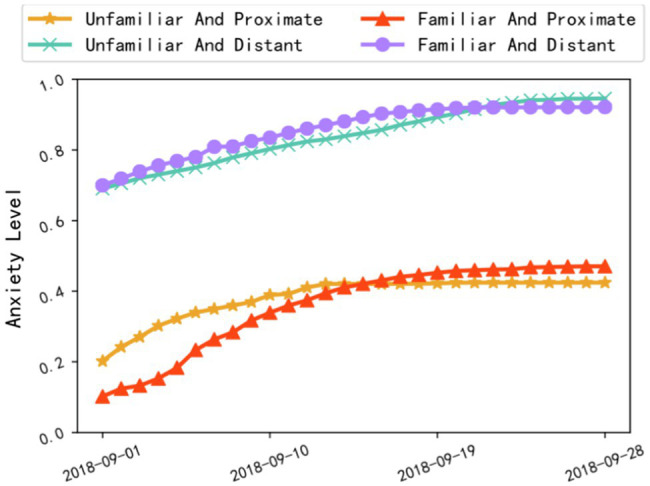
Correlation between topic (familiar/unfamiliar and proximate/distant) and collective anxiety level.

For the four types of online topic communities (whether members are familiar with the topic, and whether the topic is proximate to public interest), their anxiety showed similar overall temporal trends at different severity levels (Figure 2). In general, all communities showed a decelerating increment of collective anxiety within the observation time window. None of the collective anxiety levels reached the theoretical upper bound of the qualification for collective anxiety. In examining H1 with Pearson correlation coefficient, the topic familiarity was not significantly correlated with collective anxiety (*r*=0.126, *sig*=0.076), while the topic proximity was significantly and negatively correlated with collective anxiety (*r*=−0.352, *sig*<0.001). Therefore, H1a (topic familiarity) was not supported. For H1b (topic proximity), the more relevant (i.e., proximate) a topic is to public interest, the more it can affect the level of collective anxiety, and higher proximity to public interest correlates with lower collective anxiety. This can be explained that, with higher topic proximity to public interest, community members tend to receive more relevant information from diversified sources to better understand the topic, so that their collective emotion (including collective anxiety) may not be easily influenced, compared to otherwise. Meanwhile, whether people were familiar with a topic or not did not predict the level of collective anxiety.

*H2: Members’ declarative knowledge of the topic helps to relieve collective anxiety.*

**Figure 3:**
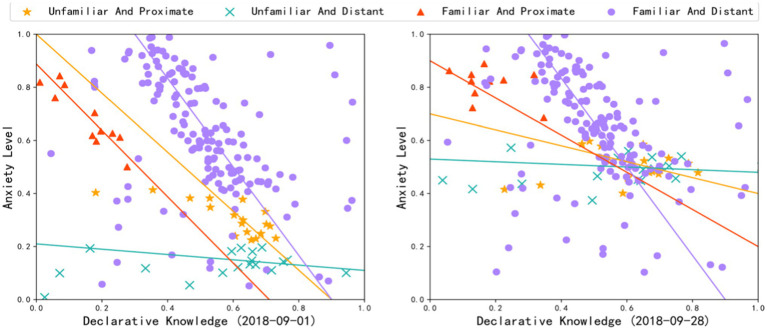
Correlation between declarative knowledge and collective anxiety level (displaying start and end points of our observation time window; other time points showed similar trends).

We computed the declarative knowledge at the community level using equation (6) and by considering the credibility of the sources of information, the similarity between messages and the topic of the community, and the readability of textual messages. The co-evolution between declarative knowledge and collective anxiety are presented in Figure 3. In examining H2, the declarative knowledge at the community level was significantly and negatively correlated with collective anxiety (R^2^=0.300, *F*=84.881, *p*<0.001, *β*=−0.833). Therefore, H2 was supported. A community in which members possess less declarative knowledge had higher collective anxiety value. In other words, we infer that members’ declarative knowledge of the topic can help to relieve collective anxiety.

*H3: A community with more influencers is more likely to have anxiety polarization.*

We tested the relation between the ratio of influencers (yellow “V” personal accounts) within each community and the standard deviation of the average daily collective anxiety. In our dataset, there was less fluctuation in the collective anxiety when there were more influential members in a community. In examining H3, the results showed that the ratio of influencers in a community was significantly and negatively correlated with the standard deviation of the average daily collective anxiety (*ρ*=−0.154, *p*<0.05). Therefore, H3 (A topic-based community containing more influential people will have lower levels of fluctuation in its anxiety) was supported, which means that, a community with more influential members exhibited less affective fluctuation, therefore, would lead to a higher level of affective polarization. In other words, for communities with higher proportion of influential members, their average collective anxiety varied less temporally. No matter the average collective anxiety is higher or lower, more influential members help to set the tone and direction of collective emotions, in our context, stabilizing the collective anxiety, and other members introduce less variations in terms of increasing or declining collective anxiety values. As a result, such situation will lead to a steadier condition where the original tendency of the group gets reinforced after deliberation, which is affective polarization.

### Regression Model to Predict Collective Anxiety

Based on the verification results of all three hypotheses, we further studied the relationship between collective anxiety as the dependent variable and following variables: topic familiarity, topic proximity, member’s competence as measured by the declarative knowledge held by a community concerning the community’s topic, frequency of interactions within a community, popularity level measured by ratio of influencers (in our study, the ratio of grass-roots users simply equals to 1 minus the ratio of influencers in the community, therefore, we used the ratio of influencers in the community to test popularity level variables), size of a community, and network level variables, i.e., density of reposting/commenting edges and diameter of a Topic-based Community. Table 3 shows our model output. Again, the more familiar members are to the community’s topic, and the more declarative knowledge they possess, the less likely the average collective anxiety increases. As we have mentioned in H1, topic familiarity does not help to predict collective anxiety levels. Echoing H2 and H3, both member’s declarative knowledge and the influencers’ ratio do have impact on the collective anxiety level. Additionally, there are no significant correlations between community size/dynamics of interactivity and the level of collective anxiety of the topic-based community.

**Table 3:** Regression output to predict collective anxiety.

Variables	*β*	SD	t	*p*
Topic familiarity	0.028	0.385	0.629	0.530
Topic proximity	−0.215^***^	0.353	−4.265	0.000
Declarative knowledge	−0.758^***^	0.168	−8.547	0.000
Ratio of influencers	−0.709^**^	0.051	−2.417	0.017
Number of posts in a Topic-based community	−8.682E-6	4134.919	−0.634	0.527
Number of comments in a Topic-based community	−5.705E-6	9843.696	−0.834	0.405
Number of members in a Topic-based community	2.935E-5	1776.189	0.904	0.367
Frequency of interactions	3.791E-7	97121.794	0.590	0.556
Density of reposting/commenting edges of a Topic-based community	0.002	10.190	0.796	0.427
Diameter of a Topic-based community	−8.057E-5	8.018	0.030	0.976
Dependent variable: average collective anxiety				

*** *Denotes statistical significance at the 1% level*.

** *Denotes statistical significance at the 5% level*.

## Discussion

### Principal Results

This paper shows the design of a computational model to explore the origins of and fluctuations in collective anxiety within an online topic-based community and the correlation between collective anxiety, topic characteristics, and the proportion of influencers in the community. It was found that the more public familiar with a topic, the more likely it was to affect the collective anxiety level and that communities whose members possessed a high level of declarative knowledge tended to have lower anxiety levels. It was also found that communities containing a higher ratio of influencers showed less fluctuation in their anxiety levels, indicating greater anxiety polarization.

The outcomes revealed a decrease in the level of collective anxiety as the proximity of the topics to the public interest increased. The most probable explanation for this trend is the community members’ exposure to information. Community members tend to search for diverse information related to the topics with high proximity of public interest. This tendency may balance their perceptions and affective attitudes toward the topics. Therefore, notwithstanding the community’s shared focus on specific topics, individual members have a greater likelihood of having their own perceptions.

The ebb and flow of topics, which is a fundamental feature of social media, may also account for the decrease in anxiety. A large number of users continuously generating and consuming content makes it more difficult for one topic to attract an inordinate amount of attention, resulting in increasingly fierce content competition (Asur et al., 2011). For the individual user, this means limited attention is allotted to a single topic. Therefore, the user’s affective focus on a particular topic may shift or disappear over time. Some scholars have focused on the connection between distraction and users’ affective states (Hodas and Lerman, 2012; Paasonen, 2016); however, an in-depth examination of the correlation between user distraction and collective anxiety is still required.

It was further revealed that the credibility of the message source impacted the affective polarization of topic-based communities. Researchers have focused on users with identity endorsement from offline social institutions in the same field; however, demonstrating sound declarative knowledge does not necessarily reflect complete understanding of an issue. The degree of anxiety polarization in a rumor-based community may be lower than in a truth-based community. Therefore, affective polarization is imparted through the user’s trust in someone, rather than through the interconnection of messages.

Although scholars have noticed affective responses to illegitimate news sources or political disinformation, they have not examined the correlation between trust and affective polarization (Barfar, 2019; Munger et al., 2020). Therefore, future research could either focus on the relationship between trust as social capital and the affective polarization of a topic-based community or examine whether the source’s credibility or the message itself is more likely to impact affective polarization.

We also hypothesized that a community with more influencers (“V” account users) would be more likely to experience anxiety polarization. Recent studies have shown that an iterative relationship exists between polarization and the influence of online celebrities (Garibay et al., 2019). As a result, messages from “V” account users are more visible than messages from other users. This inequality warrants reflection and requires intervention from policymakers and developers. Additionally, the Weibo verification of online influencers cannot adequately reflect the interaction between influencers.

An online topic-based community is a multi-layered network where diverse interactive patterns are encouraged (Mittal and Bhatia, 2019). In particular, “V” account users are more likely to communicate with their “friends” than with their followers. They are more frequently mentioned but do not actively repost or reply to their followers. Weibo measures a user’s influence by one-way searching or following, neglecting the user’s sociability, even though sociability should also be considered a significant factor in a user’s level and the subsequent ranking of the user’s content (Shin and Lee, 2012). For our study, when we did not distinguish between influencers and ordinary users, interactivity had no significant effect on collective anxiety. But this does not necessarily mean the interactivity among influencers has no significant impact on collective anxiety. Based on our observation, conversely, the forwarding between influencers may have led to a drastic increase in the original message’s emotional impact. Therefore, the interaction between influencers in a topic-based community may be another variable correlated with affective polarization that is worthy of further examination.

The existing scholarly discourse on affective polarization on social media is inadequate in three respects. The first of these pertains to the capacity to treat emotion as an abstract phenomenon by focusing squarely on arbitrary directions instead of understanding more tangible emotions, including anxiety. Additionally, investigating unilateral causes of affective polarization while refusing to acknowledge the nexus between causality is myopic. Third, devoting particular attention to discrete cases rather than designing general computing tools is misguided. This paper aims to fill these gaps by providing a more comprehensive computing model to gauge the nature of and reason for the fluidity underlying collective anxiety.

Some researchers consider anxiety to be a robust mobilizer of the public (Marcus and MacKuen, 1993; Brader et al., 2008; Valentino et al., 2008; Beam et al., 2018). This paper echoes this view by highlighting anxiety as a discrete emotion and further quantifying it at the community level. Specifically, it provides a machine-learning model that computes changes to collective anxiety and facilitates a depiction of the latter’s tendencies. It uses a bimodal network to evaluate individual anxiety by utilizing both online profiles and social network messages, giving a more comprehensive evaluation for individuals. By considering anxiety propagation among individuals, it uses a cascading model to produce the collective anxiety score at a community level, rather than simply averaging the individual anxiety scores. It also seeks to understand the connection between the causes and consequences of affective polarization.

### Limitations and Future Directions

By focusing on affective polarization, mediated by Weibo, this paper attempts to address this context-specific gap in the literature. Future work may focus on the dynamics between collective-anxiety and interactivity across platforms. In this way, it may be possible to compare findings and determine whether the community member’s collective anxiety would be more balanced when concerning the mix of affective polarization in cross-platform interactivity (Del Vicario et al., 2016; Beam et al., 2018; Harel et al., 2020) on the same topic. The potential for ethnicity distribution and interactions among influencers that may impact the platform’s collective anxiety level is also worth investigating in the future. Considering continuity with current research, we will focus on how the community’s influencers’ interactivity might impact the affective polarization. A scholar insightfully reminded us that users’ perceptions of transparency are a pivotal element of the trust–judgment process (Shin, 2019). Specific to our research, influencers’ interactions in the topic-based community are visible to all group members. It will be meaningful to examine if this kind of visibility contributes to other users’ perception of transparency and further impact the dynamics between the in-group trust and its affective polarization.

Second, the data may not represent all Weibo users as short-term longitudinal studies can be influenced by several factors that influence tendencies and topic selection. For example, as researchers could not interact with the shared content, they could not determine if individuals without accounts were perusing a particular online topic. Besides, researchers, to some extent, cannot determine whether a user’s anxiety is borne out of their authenticity or not, given that people’s social media performance is much about what Goffman (1959) called “self-presentation”. Therefore, we treat the data of this paper in an effect-oriented way. We care more about the data they have presented on social media, which truly influenced others’ anxiety and further driven their online behavior in a topic-based community. Future in-depth interviews are needed to collect more of the users’ won life stories and experiences.

Third, as authors suggest that anxiety values change at different junctures based on the degree of affective polarization, the implication is that users who were not actively engaged in the discussion at the time of the study would be eliminated from the analysis. In the future, both questionnaires and offline interviews should be utilized to derive hypotheses with stronger conviction. With almost every account controlled by a particular individual, it is essential to continue examining if silent users also affect the degree of affective polarization.

## Conclusion

The manner in which anxiety on social media polarizes warrants further examination. Contemporary research suggests this anxiety exists at the individual level but the implications for the collective have not been thoroughly explored. This paper examines the origin and change of collective anxiety in topic-based communities on Weibo. Authors found the collective anxiety to augment due to topic proximities to public interest and members’ lack of declarative knowledge on topics, while to decline with an increasing portion of online influencers. These findings indicate that anxiety is induced due to a lack of credibility. Also, the amount of conflicting information shared by different people places them in a state of flux. Therefore, a community with more influencers may be more likely to experience anxiety polarization, bringing forth the issue of layered information and inequality. The framework we proposed and the results in our empirical study demonstrate the feasibility of quantitative evaluation of collective anxiety and its polarization. Our findings can be utilized in further endeavors seeking to understand the development and effects of online collective affection.

## Data Availability Statement

The raw data supporting the conclusions of this article will be made available by the authors, without undue reservation.

## Ethics Statement

The studies involving human participants were reviewed and approved by the Research Ethics Committee of Renmin University of China. The patients/participants provided their written informed consent to participate in this study.

## Author Contributions

All authors listed have made a substantial, direct and intellectual contribution to the work, and approved it for publication.

## Funding

This research is supported by the National Natural Science Foundation of China under Grant No. 61802414, the Journalism and Marxism Research Center, Renmin University of China under Grant No. MXG201907, the Social Science Foundation of Beijing under Grant No. 18XCC011, and the Humanities and Social Sciences Base Foundation of Ministry of Education of China under Grant No. 16JJD860008.

## Conflict of Interest

The authors declare that the research was conducted in the absence of any commercial or financial relationships that could be construed as a potential conflict of interest.

## Publisher’s Note

All claims expressed in this article are solely those of the authors and do not necessarily represent those of their affiliated organizations, or those of the publisher, the editors and the reviewers. Any product that may be evaluated in this article, or claim that may be made by its manufacturer, is not guaranteed or endorsed by the publisher.

## Footnotes

^1^In practice, the Self-Rating Anxiety Scales (SAS) can be used to evaluate a person’s anxiety level. The SAS used in this study can be found at https://github.com/public-anxiety/data/blob/master/SAS(EN).doc

^2^In an online social network, if two users are connected by k-1 numbers of other users, they form each other’s k^th^ connection (k ≥ 1). For example, users u_1_ and u_2_ are direct friends online, as shown by the first-degree connections to each other. If u_3_ is a direct friend of u_2_ but not u_1_, then u_3_ is the second-degree connection for u_1_.

## References

[ref1] Asur S. Huberman B. A. Szabo G. Wang C. (2011). Trends in Social Media: Persistence and Decay. Available at: http://arxiv.org/abs/1102.1402 (Accessed: September 15, 2021).

[ref2] Barfar A. (2019). Cognitive and affective responses to political disinformation in Facebook. Comput. Hum. Behav. 101, 173–179. doi: 10.1016/j.chb.2019.07.026

[ref3] Bar-Tal D. Halperin E. Rivera J. D. (2007). Collective emotions in conflict situations: societal implications. J. Soc. Issues 63, 441–460. doi: 10.1111/j.1540-4560.2007.00518.x

[ref4] Beam M. A. Hutchens M. J. Hmielowski J. D. (2018). Facebook news and (de)polarization: reinforcing spirals in the 2016 US election. Inf. Commun. Soc. 21, 940–958. doi: 10.1080/1369118X.2018.1444783

[ref5] Berelson B. R. Lazarsfeld P. F. McPhee W. N. (1954). Voting: Study of Opinion Formation in a Presidential Campaign. Chicago: University of Chicago Press.

[ref6] Bessi A. Zollo F. Vicario M. D. Puliga M. Scala A. Caldarelli G.. (2016). Users polarization on Facebook and Youtube. PLoS One 11:e0159641. doi: 10.1371/journal.pone.0159641, PMID: 27551783 PMC4994967

[ref7] Blackman L. (2012). Immaterial Bodies: Affect, Embodiment, Mediation. Thousand Oaks, California: SAGE.

[ref8] Blevins J. L. Lee J. J. McCabe E. E. Edgerton E. (2019). Tweeting for social justice in #Ferguson: affective discourse in twitter hashtags. New Media Soc. 21, 1636–1653. doi: 10.1177/1461444819827030

[ref9] Bon G. L. (2002). The Crowd: A Study of the Popular Mind. New York: Dover Publications.

[ref10] Borgatti S. P. Everett M. G. Johnson J. C. (2013). Analyzing Social Networks. London: SAGE Publications Ltd.

[ref11] Brader T. Valentino N. A. Suhay E. (2008). What triggers public opposition to immigration? Anxiety, group cues, and immigration threat. Am. J. Polit. Sci. 52, 959–978. doi: 10.1111/j.1540-5907.2008.00353.x

[ref12] China Internet Network Information Center (2017). Research Report on 2016 user behavior of social applications in China. Available at: http://www.cnnic.cn/hlwfzyj/hlwxzbg/sqbg/201712/P020180103485975797840.pdf (Accessed June 15, 2021).

[ref13] China Internet Network Information Center (2019). The 43rd China Statistical Report on Internet Development. Available at: http://www.cnnic.cn/hlwfzyj/hlwxzbg/hlwtjbg/201902/P020190318523029756345.pdf (Accessed June 15, 2021).

[ref14] Colleoni E. Rozza A. Arvidsson A. (2014). Echo chamber or public sphere? Predicting political orientation and measuring political homophily in twitter using big data: political homophily on twitter. J. Commun. 64, 317–332. doi: 10.1111/jcom.12084

[ref15] Curiel C. P. (2020). Political influencers/leaders on twitter. An analysis of the Spanish digital and media agendas in the context of the Catalan elections of 21 December 2017. KOME − Int. J. Pure 8, 88–108. doi: 10.17646/kome.75672.46

[ref16] Dahlgren P. (2005). The internet, public spheres, and political communication: dispersion and deliberation. Polit. Commun. 22, 147–162. doi: 10.1080/10584600590933160

[ref17] Davenport T. H. (2000). Working Knowledge: How Organizations Manage What they Know. Boston: Harvard Business School Press.

[ref18] Del Vicario M. Vivaldo G. Bessi A. Zollo F. Scala A. Caldarelli G.. (2016). Echo chambers: emotional contagion and group polarization on Facebook. Sci. Rep. 6:37825. doi: 10.1038/srep37825, PMID: 27905402 PMC5131349

[ref19] Eliacik A. B. Erdogan N. (2018). Influential user weighted sentiment analysis on topic based microblogging community. Expert Syst. Appl. 92, 403–418. doi: 10.1016/j.eswa.2017.10.006

[ref20] Erickson B. H. (1988). “The relational bias of attitudes,” in Social Structures: A Network Approach. eds. Wellman B. Berkowitz S. D. (New York: Oxford University Press), 99–121.

[ref21] Festinger L. (1954). A theory of social comparison processes. Hum. Relat. 7, 117–140. doi: 10.1177/001872675400700202

[ref22] Garibay I. Mantzaris A. V. Rajabi A. Taylor C. E. (2019). Polarization in social media assists influencers to become more influential: analysis and two inoculation strategies. Sci. Rep. 9:18592. doi: 10.1038/s41598-019-55178-8, PMID: 31819120 PMC6901574

[ref23] Garrett K. N. Bankert A. (2020). The moral roots of partisan division: how moral conviction heightens affective polarization. Br. J. Polit. Sci. 50, 621–640. doi: 10.1017/S000712341700059X

[ref24] Garrett R. K. Gvirsman S. D. Johnson B. K. Tsfati Y. Neo R. Dal A. (2014). Implications of pro- and counter-attitudinal information exposure for affective polarization. Hum. Commun. Res. 40, 309–332. doi: 10.1111/hcre.12028

[ref25] Goffman E. (1959). The Presentation of Self in Everyday Life (Doubleday Anchor Books). Garden City, NY: Doubleday.

[ref26] González-Ibáñez R. I. Shah C. (2010). “Group’s Affective Relevance: A Proposal for Studying Affective Relevance in Collaborative Information Seeking.” in Proceedings of the 16th ACM International Conference on Supporting group work. November 07, 2010, 317–318.

[ref27] Gross K. (2008). Framing persuasive appeals: episodic and thematic framing, emotional response, and policy opinion. Polit. Psychol. 29, 169–192. doi: 10.1111/j.1467-9221.2008.00622.x

[ref28] Hadley C. Patil C. L. (2008). Seasonal changes in household food insecurity and symptoms of anxiety and depression. Am. J. Phys. Anthropol. 135, 225–232. doi: 10.1002/ajpa.20724, PMID: 18046777

[ref29] Harel T. O. Jameson J. K. Maoz I. (2020). The normalization of hatred: identity, affective polarization, and dehumanization on Facebook in the context of intractable political conflict. Social Media + Society 6:2056305120913983. doi: 10.1177/2056305120913983

[ref30] Hodas N. O. Lerman K. (2012). “How Visibility and Divided Attention Constrain Social Contagion.” in 2012 International Conference on Privacy, Security, Risk and Trust and 2012 International Confernece on Social Computing. September 3–5, 2012, 249–257.

[ref31] Hopkins N. Reicher S. D. Khan S. S. Tewari S. Srinivasan N. Stevenson C. (2016). Explaining effervescence: investigating the relationship between shared social identity and positive experience in crowds. Cognit. Emot. 30, 20–32. doi: 10.1080/02699931.2015.1015969 25787295 PMC4704436

[ref32] Hutchens M. J. Hmielowski J. D. Beam M. A. (2019). Reinforcing spirals of political discussion and affective polarization. Commun. Monogr. 86, 357–376. doi: 10.1080/03637751.2019.1575255

[ref33] Hwang H. Kim Y. Huh C. U. (2014). Seeing is believing: effects of uncivil online debate on political polarization and expectations of deliberation. J. Broadcast. Electron. Media 58, 621–633. doi: 10.1080/08838151.2014.966365

[ref34] Iyengar S. Lelkes Y. Levendusky M. Malhotra N. Westwood S. J. (2019). The origins and consequences of affective polarization in the United States. Annu. Rev. Polit. Sci. 22, 129–146. doi: 10.1146/annurev-polisci-051117-073034

[ref35] Iyengar S. Sood G. Lelkes Y. (2012). Affect, not ideology: a social identity perspective on polarization. Public Opin. Q. 76, 405–431. doi: 10.1093/poq/nfs038

[ref36] Jo W. Lee J. Park J. Kim Y. (2020). Online information exchange and anxiety spread in the early stage of the novel coronavirus (COVID-19) outbreak in South Korea: structural topic model and network analysis. J. Med. Internet Res. 22:e19455. doi: 10.2196/19455, PMID: 32463367 PMC7268668

[ref37] Johnston J.. (2017). Whose Interests? Why Defining the ‘Public Interest’ is Such a Challenge, the CONVERSATION, Available at: https://theconversation.1363com/whose-interests-why-defining-the-public-interest-is-such-a-1364 challenge-84278(Accessed: September 4, 2021)ss.

[ref38] Kardara M. Papadakis G. Papaoikonomou T. Tserpes K. Varvarigou T. (2012). “Influence Patterns in Topic Communities of Social Media.” in Proceedings of the 2nd International Conference on Web Intelligence, Mining and Semantics. June 13, 2012, 1–12.

[ref39] Krippendorff K. (2004). Reliability in content analysis: Some common misconceptions and recommendations. Hum. Commun. Res. 30, 411–433. doi: 10.1111/j.1468-2958.2004.tb00738.x

[ref40] Lau R. R. Andersen D. J. Ditonto T. M. Kleinberg M. S. Redlawsk D. P. (2017). Effect of media environment diversity and advertising tone on information search, selective exposure, and affective polarization. Polit. Behav. 39, 231–255. doi: 10.1007/s11109-016-9354-8

[ref41] Lazarsfeld P. F. Merton R. K. (1954). “Friendship as a social process: A substantive and methodological analysis”, in Freedom and Control in Modern Society. eds. Berger M. Abel T. Page C. H. (New York: Van Nostrand) 18–66.

[ref42] Lee F. L. F. (2016). Impact of social media on opinion polarization in varying times. Communication and the Public 1, 56–71. doi: 10.1177/2057047315617763

[ref43] Leeper T. J. (2014). The informational basis for mass polarization. Public Opin. Q. 78, 27–46. doi: 10.1093/poq/nft045

[ref44] Lelkes Y. Westwood S. J. (2017). The limits of partisan prejudice. J. Polit. 79, 485–501. doi: 10.1086/688223

[ref45] Levendusky M. S. (2018). Americans, not partisans: can priming American National Identity Reduce Affective Polarization? J. Polit. 80, 59–70. doi: 10.1086/693987

[ref46] Levy D. A. Nail P. R. (1993). Contagion: a theoretical and empirical review and reconceptualization. Genet. Soc. Gen. Psychol. Monogr. 119, 233–284. PMID: 8405969

[ref47] Lu Y. Lee J. K. (2019). Partisan information sources and affective polarization: panel analysis of the mediating role of anger and fear. J. Mass Commun. Q. 96, 767–783. doi: 10.1177/1077699018811295

[ref48] Marcus G. E. MacKuen M. B. (1993). Anxiety, enthusiasm, and the vote: the emotional underpinnings of learning and involvement during presidential campaigns. Am. Polit. Sci. Rev. 87, 672–685. doi: 10.2307/2938743

[ref49] Mäs M. Flache A. Takács K. Jehn K. A. (2013). In the short term we divide, in the long term we unite: demographic crisscrossing and the effects of faultlines on subgroup polarization. Organ. Sci. 24, 716–736. doi: 10.1287/orsc.1120.0767

[ref50] May R. (2015). The Meaning of Anxiety. San Francisco: Hauraki Publishing.

[ref51] McLaughlin B. Holland D. Thompson B. A. Koenig A. (2020). Emotions and affective polarization: how enthusiasm and anxiety about presidential candidates affect interparty attitudes. Am. Politics Res. 48, 308–316. doi: 10.1177/1532673X19891423

[ref52] Meppelink C. S. Smit E. G. Fransen M. L. Diviani N. (2019). “I was right about vaccination:” confirmation bias and health literacy in online health information seeking. J. Health Commun. 24, 129–140. doi: 10.1080/10810730.2019.1583701, PMID: 30895889

[ref53] Mittal R. Bhatia M. P. S. (2019). Classifying the influential individuals in multi-layer social networks. IJECME 8, 21–32. doi: 10.4018/IJECME.2019010102

[ref54] Moscovici S. Zavalloni M. (1969). The group as a polarizer of attitudes. J. Pers. Soc. Psychol. 12, 125–135. doi: 10.1037/h0027568

[ref55] Munger K. Luca M. Nagler J. Tucker J. (2020). The (null) effects of Clickbait headlines on polarization, trust, and learning. Public Opin. Q. 84, 49–73. doi: 10.1093/poq/nfaa008

[ref56] Myers D. G. Lamm H. (1975). The polarizing effect of group discussion: The discovery that discussion tends to enhance the average prediscussion tendency has stimulated new insights about the nature of group influence. Am. Sci. 63, 297–303. PMID: 1147368

[ref57] Myers S. A. Sharma A. Gupta P. Lin J. (2014). “Information Network or Social Network? The Structure of the Twitter Follow Graph.” in Proceedings of the 23rd International Conference on World Wide Web. April 07, 2014, 493–498.

[ref58] Nabi R. L. (2010). The case for emphasizing discrete emotions in communication research. Commun. Monogr. 77, 153–159. doi: 10.1080/03637751003790444

[ref59] Nakayachi K. Yokoyama H. M. Oki S. (2015). Public anxiety after the 2011 Tohoku earthquake: fluctuations in hazard perception after catastrophe. J. Risk Res. 18, 156–169. doi: 10.1080/13669877.2013.875936

[ref60] Olofsson J. K. (2010). Mass movements in computer-mediated environments. Inf. Commun. Soc. 13, 765–784. doi: 10.1080/13691180903521539

[ref61] Paasonen S. (2016). Fickle focus: distraction, affect and the production of value in social media. First Monday 21. Available at: http://firstmonday.org/ojs/index.php/fm/article/view/6949 (Accessed: 25 May 2017).

[ref62] Pariser E. (2011). The Filter Bubble. New York: Penguin Press.

[ref63] Pierce J. (1970). Party identification and the changing role of ideology in American politics. Midwest Journal of Political Science 14, 25–42. doi: 10.2307/2110391

[ref64] Reinikainen H. Munnukka J. Maity D. Luoma-aho V. (2020). ‘You really are a great big sister’ – parasocial relationships, credibility, and the moderating role of audience comments in influencer marketing. J. Mark. Manag. 36, 279–298. doi: 10.1080/0267257X.2019.1708781

[ref65] Santabárbara J. Lasheras I. Lipnicki D. M. Bueno-Notivol J. Pérez-Moreno M. López-Antón R.. (2021). Prevalence of anxiety in the COVID-19 pandemic: an updated meta-analysis of community-based studies. Prog. Neuro-Psychopharmacol. Biol. Psychiatry 109:110207. doi: 10.1016/j.pnpbp.2020.110207, PMID: 33338558 PMC7834650

[ref66] Schraw G. (2006). “Knowledge: structures and process,” in Handbook of Educational Psychology. 2nd *Edn*. *Vol*. 2006. eds. Alexander P. A. Winne P. H. (Mahwah, NJ: Lawrence Erlbaum), 245–264.

[ref67] Schweitzer F. Garcia D. (2010). An agent-based model of collective emotions in onlinecommunities. Eur. Phys. J. B 77, 533–545. doi: 10.1140/epjb/e2010-00292-1

[ref68] Shahini-Hoxhaj R. (2018). Facebook and political polarization: an analysis of the social media impact on the Kosovo-Serbia dialogue. J. Media Res. 11, 71–93. doi: 10.24193/jmr.32.6

[ref501] Shin D. (2016). Cross-Platform Users’ Experiences Toward Designing Interusable Systems. Int. J. Hum-Comput. Int. 32, 503–514. doi: 10.1080/10447318.2016.1177277

[ref69] Shin D. (2019). Blockchain: The emerging technology of digital trust. Telematics Inform. 45:101278. doi: 10.1016/j.tele.2019.101278

[ref502] Shin D. Hwang Y. (2020). The effects of security and traceability of blockchain on digital affordance. Online Inf. Rev. 44, 913–932. doi: 10.1108/OIR-01-2019-0013

[ref70] Shin D. Kim J. (2015). Social viewing behavior in social TV: proposing a new concept of socio-usability. Online Inf. Rev. 39, 416–434. doi: 10.1108/OIR-12-2014-0299

[ref71] Shin H. Lee J. (2012). Impact and degree of user sociability in social media. Inf. Sci. 196, 28–46. doi: 10.1016/j.ins.2012.01.040

[ref72] Soares F. B. Recuero R. Zago G. (2018). “Influencers in Polarized Political Networks on Twitter.” in Proceedings of the 9th International Conference on Social Media and Society. July 18, 2018, 168–177.

[ref73] Sokolova K. Kefi H. (2020). Instagram and YouTube bloggers promote it, why should I buy? How credibility and parasocial interaction influence purchase intentions. J. Retail. Consum. Serv. 53:101742. doi: 10.1016/j.jretconser.2019.01.011

[ref74] Stage C. (2013). The online crowd: a contradiction in terms? On the potentials of Gustave Le Bon’s crowd psychology in an analysis of affective blogging. Distinktion 14, 211–226. doi: 10.1080/1600910X.2013.773261

[ref75] Sung Y.-T. Chang T.-H. Lin W.-C. Hsieh K.-S. Chang K.-E. (2016). CRIE: an automated analyzer for Chinese texts. Behav. Res. Methods 48, 1238–1251. doi: 10.3758/s13428-015-0649-1, PMID: 26424442

[ref76] Sunstein C. R. (2002). The law of group polarization. J. Polit. Philos. 10, 175–195. doi: 10.1111/1467-9760.00148

[ref77] Sunstein C. R. (2009). Republic.com 2.0 (1. pbk. print). Princeton and Oxford: Princeton University Press.

[ref78] Ta N. Li K. Yang Y. Jiao F. Tang Z. Li G. (2020). Evaluating public anxiety for topic-based communities in social networks. IEEE Trans. Knowl. Data Eng. 1. doi: 10.1109/TKDE.2020.2989759

[ref79] Takebayashi Y. Lyamzina Y. Suzuki Y. Murakami M. (2017). Risk perception and anxiety regarding radiation after the 2011 Fukushima nuclear power plant accident: a systematic qualitative review. Int. J. Environ. Res. Public Health 14:1306. doi: 10.3390/ijerph14111306, PMID: 29077045 PMC5707945

[ref80] Tan C. Lee L. Tang J. Jiang L. Zhou M. Li P. (2011). “User-Level Sentiment Analysis Incorporating Social Networks.” in Proceedings of the 17th ACM SIGKDD International Conference on Knowledge Discovery and Data Mining. August 21, 2011, 1397–1405.

[ref81] Tyagi A. Uyheng J. Carley K. M. (2020). Affective Polarization in Online Climate Change Discourse on Twitter. Available at: http://arxiv.org/abs/2008.13051 (Accessed: September 15, 2021).

[ref82] Valentino N. A. Hutchings V. L. Banks A. J. Davis A. K. (2008). Is a worried citizen a good citizen? Emotions, political information seeking, and learning via the internet. Polit. Psychol. 29, 247–273. doi: 10.1111/j.1467-9221.2008.00625.x

[ref83] Veirman M. D. Cauberghe V. Hudders L. (2017). Marketing through Instagram influencers: the impact of number of followers and product divergence on brand attitude. Int. J. Advert. 36, 798–828. doi: 10.1080/02650487.2017.1348035

[ref84] Vilanova F. Beria F. M. Costa Â. B. Koller S. H. (2017). Deindividuation: from Le Bon to the social identity model of deindividuation effects. Cogent Psychol. 4:1308104. doi: 10.1080/23311908.2017.1308104

[ref85] Webster S. W. Abramowitz A. I. (2017). The ideological foundations of affective polarization in the U.S. electorate. Am. Politics Res. 45, 621–647. doi: 10.1177/1532673X17703132

[ref86] Weibo Data Center (2019). Report on the Development of Weibo Users in 2018. Available at: https://data.weibo.com/report/reportDetail?id=433&display=0&retcode=6102. (Accessed June 15, 2021).

[ref87] Wojcieszak M. Winter S. Yu X. (2020). Social norms and selectivity: effects of norms of open-mindedness on content selection and affective polarization. Mass Commun. Soc. 23, 455–483. doi: 10.1080/15205436.2020.1714663

[ref88] Xu S. Zhou A. (2020). Hashtag homophily in twitter network: examining a controversial cause-related marketing campaign. Comput. Hum. Behav. 102, 87–96. doi: 10.1016/j.chb.2019.08.006

[ref89] Zhao Y. Liu J. Tang J. Zhu Q. (2013). Conceptualizing perceived affordances in social media interaction design. ASLIB Proc. 65, 289–303. doi: 10.1108/00012531311330656

